# Role of hormones in hypoactive sexual desire disorder and current treatment

**DOI:** 10.4274/jtgga.2017.0071

**Published:** 2017-12-15

**Authors:** Ahmed AlAwlaqi, Houda Amor, Mohamed E. Hammadeh

**Affiliations:** 1 Department of Obstetrics and Gynaecology, University of Saarland, Homburg, Germany

**Keywords:** Hyposexuality, hormone, women, menopause, hypoactive

## Abstract

Over the decades, female sexual dysfunction (FSD) has grown to be an increasingly potential problem that complicates the quality of life among women. In the current review, FSD refers to recurrent and persistent problems with sexual orgasm, desire, or response. One of the most common subtypes of FSD that has evoked increased research interest in the scientific community is hyposexuality. Today, there is a consensus that hyposexuality is a multifactorial condition that manifests with reduced sexual desire resulting in significant interpersonal distress. The objective of the current review was to examine how hormonal profile triggers propagate hypoactive sexual desire disorder (HSDD), and to highlight effective treatment interventions that can be used to manage the condition. The current review describes HSDD as a sexual dysfunction characterized by the absence or lack of sexual desire and fantasies for sexual activities. The review argues that even if the role of sexual hormones is essential in modulating HSDD through therapeutic interventions, an effective comprehension of the biologic mechanisms underlying HSDD is necessary. There is a consensus in the literature that HSDD still poses significant challenges due to the lack of properly formulated treatment regimens and absence of clear clinical guidelines. That is, a better intervention consisting of both psycho-relational and biologic aspects is compulsory if tailored management and accurate diagnosis of HSDD in clinical practice are to be realised. The review concludes that, to date, a reliable clinical intervention to manage hyposexuality is still absent and more interventions, in terms of safety and efficacy, are required. Thus, additional investigation is required to document precise hormonal or non-hormonal pharmacotherapeutic agents for individualised care among patients with HSDD.

## INTRODUCTION

The problem of low sexual desire affects women of all ages, which contributes to potential negative outcomes including reduced well-being and quality of life (1,2). Over the years, low sexual desire has been widely regarded as part of broader female sexual dysfunction (FSD) conditions (3), of which HSDD is more prevalent (4,5). According to the American Psychiatric Association’s Diagnostic and Statistical Manual of Mental Disorders (DSM-IV), HSDD is defined as a persistent absenteeism of sexual craving for sexual activities (6). However, the International Classification of Disease by the World Health Organization (7) and the DSM-IV tool (6) have reached a consensus that the definition of HSDD must include several aspects of accurate diagnosis. These include the presence of interpersonal difficulties and/or personal distress, in addition to the lack of sexual desires or fantasies for sex-related activities (6,7). A similar claim has also been supported by the American Foundation for Urologic Disease, on the basis that both sexually-related individual distress and low sexual desire should be observed for a person to be positively diagnosed as having HSDD (7,8).

Often, when cases of low sexual desire are reported, the most common diagnosis is assumed to be generalised acquired HSDD. HSDD is mostly not reliant on a specific situation, and often develops at a time when the desire for sex is assumed to be ordinary (8). As such, the presence of HSDD may manifest as a comorbidity in addition to a dysfunctional sexual experience, even if no exclusive connection can be made with the physiologic effects of a therapeutic agent or medical conditions (9). Recently, the International Consultation on Sexual Medicine (10) recommended the need to redefine HSDD because of the diverse heterogeneity among women and their sexual responses. As such, according to Sand and Fisher (11), a new definition for HSDD is set to be taken into consideration in the upcoming DSM-V.

Today, the aetiology of HSDD has not been holistically agreed upon, although scholars and researchers agree that the condition is multifactorial (12). To elaborate, HSDD has been elucidated to be triggered by factors such as psychiatric issues (12), behavioural components (13), and neuroendocrine changes (14,15). Previous studies largely centred on understanding how biologic and behavioural aspects contribute to HSDD, with a primary focus on assessment tools; the use of hormonal assays and validated behavioural questionnaires (12). Irrespective of their use, however, these methods have not completely helped in resolving the puzzle and yielding satisfactory elaboration for the development and cause of FSD conditions, and specifically HSDD.

The next section discusses how ageing factors, such as menopause, are associated with HSDD. Second, the correlation between hormonal profile and HSDD will be detailed, taking into account medical factors that can result in a hormonal imbalance. Third, the psychological and psychosocial factors and their effect on HSDD are also outlined. Fourth, the current treatment plans for HSDD are discussed before offering concluding remarks on the current review issue.

## AGEING FACTORS, MENOPAUSE, AND HSDD

Despite the current consensus in the literature that FSD can manifest at any age in a woman’s life, researchers such as Sarrel (16) documented that during menopause, up to 40% of women experience reduced sexual libido. Moreover, this claim has been supported by a survey (17) undertaken on 31,581 women aged 18 years and above in the United States of America. The study found that the higher prevalence of HSDD was in women above the age of 45 years, and distress was reported to be a major concern among younger women (12.3%) compared with older women (7.4%) aged ≥65 years.

**Figure 1:**
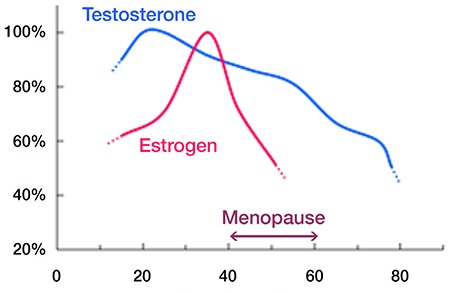
Hormone production before and after menopausal years (19)

Although sexuality is essential to both young and older women, lack of a satisfying sexual life negatively impacts on the overall quality of life (18). The trend is particularly reflected among female groups that experience an unexpected rapid decline in hormone levels as a result of chemical menopause or even post-surgical events. Figure 1 shows hormone production as a function of age, both before and after menopause (19). As evident, between the age of 20 and 40 years, there is an increase in the production of sex hormones, before a gradual decline is experienced during menopause and post-menopause years of 45 years and above.

On the contrary, other scholars argue that based on longitudinal findings, relationship issues and other non-biologic factors can strongly impact on the overall sexual experience of women other than menopausal changes alone (20). For example, research from the Massachusetts Women’s Health Survey reported that the onset of menopause contributes to reduced sexual desire. Nonetheless, anxiety, depression, and other relationship changes including conflict in the family, the condition of the relationship, sexual function, and health of a partner can contribute to substantial FSD (21). The common assumption is that menopause contributes to reduced sexual desire as a result of low production of hormones from the ovaries, resulting in loss of oestrogen and reduction in testosterone. The next subsections elaborate on the relationship between low testosterone and oestrogen levels on HSDD.

## LOW TESTOSTERONE AND HSDD

**Figure 2:**
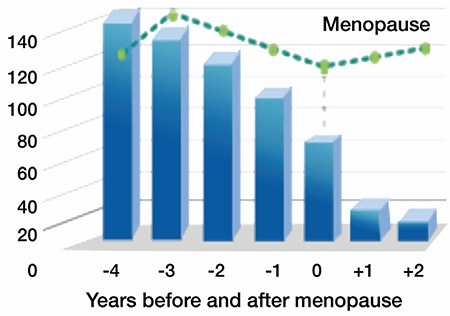
The change in testosterone levels during menopause (24)

Scholars have reported that low production of testosterone plays a central role in HSDD. One of the key reasons in support of this claim is that testosterone initiates sexual activities and proliferates sexual desire and behaviour. In addition, testosterone is essential in modulating clitoral and vaginal physiology to facilitate genital lubrication, sensation, and engorgement (22). Therefore, a lack of testosterone has been reported to contribute to low libido and to reduced sexual pleasure and receptivity (23). Also, low levels of testosterone have been correlated with lack of sexual motivation, fatigue, distress, and overall reduce the sense of well-being (24). Figure 2 shows that there is a significant decline in the production of testosterone four years before menopause, during menopause, and two years into menopause.

It is not unusual for women in their pre-menopausal years with functional ovulatory cycles to report HSDD. One of the main causes of such reduced sexual expression can be attributed to low levels of testosterone, which start to reduce in the mid-30s among women and continue to reduce at a constant rate of about 50% of their highest levels by the time they reach menopause. A recent report on women’s sexuality and health found that young women who had undergone surgical procedures reported high levels of HSDD resulting from the effects of bilateral oophorectomy where both ovaries are removed. Such procedures have been noted to contribute to about 50% reduction in the levels of testosterone (25). Hence, there seems to be a close relationship between the production of testosterone and reduced sexual desire, with more effects felt among older women in their post-menopause years and women who have undergone oophorectomy compared with younger ladies and those in their premenopausal years (26).

**Figure 3:**
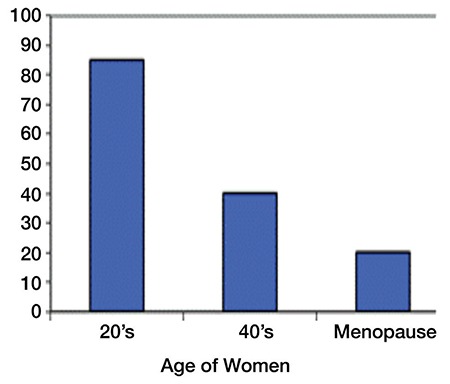
Declining levels of testosterone in women (26)

Figure 3 further shows that with increasing age, the levels of testosterone reduce and by the time a woman reaches menopause, the levels of testosterone are almost a quarter of what they were in their early 20s. According to Simon et al. (26), such a severe reduction in testosterone levels makes women gain weight, feel depressed and tired, and completely blocks most of their sex drive.

## LOW OESTROGEN LEVELS AND HSDD

**Figure 4:**
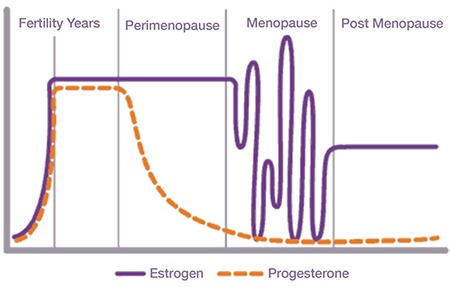
The variation in production of oestrogen and progesterone hormones throughout the female’s life (30)

Besides low testosterone levels, low sex drive among women can also be affected by reduced levels of oestrogen during postmenopausal years. Low levels of oestrogen results in vulvovaginal dryness and atrophy in addition to initiating changes of genital function through reduced sensory perception and decreased clitoral blood flow (27). As such, it becomes apparent that lack of oestrogen is associated with vaginal discomfort due to dryness and genital insensitivity, making it difficult for an individual to actively respond to sexual expression and cues, considering a reduced impact on desire (28). Researchers have recommended the use of oestrogen therapies to treat dyspareunia and vaginal dryness resulting from vulvovaginal atrophy (28). However, oestrogen-based therapies have been questioned as to whether they contribute to the effect after precise use in managing low sexual desire, in the event that low sexual events results from issues such as loss of genital pleasure, sensation, or as a consequence of pain (29). Figure 4 shows the variation in oestrogen production during years of fertility, perimenopause, menopause, and post-menopause.

As evident from Figure 4, there is a high variation in oestrogen production during menopause, and these fluctuations levels contribute to decreased sex libido among women. Besides, both peri- and post-menopausal individuals can experience HSDD due to low levels or deficiency in oestrogen hormone production (30,31). Laumann et al. (32) argued that menopause results when the levels of circulating oestrogen reduce, and this reduction leads to vaginal dryness, painful intercourse (dyspareunia), and inability to lubricate. In this case, oral oestrogen therapy is often recommended as a replacement to relieve mood changes, hot flashes, and alleviate irregular sleep patterns and improve the quality of life among women (33,34,35).

However, a study by Laumann et al. (36) on sexual dysfunction among American women reported that even if oestrogen replacement could assist in treating the symptoms linked to menopause, it would potentially have negative impacts on the levels of testosterone and further lead to HSDD. One of the reasons for this is that oral oestrogen can increase the levels of circulating sex hormone-binding globulin (SHBG) among menopausal women (37,38,39). O elaborate, SHBG has been reported as a protein that can bind testosterone and as a result, lead to lowering of free testosterone levels in the blood (40). Therefore, if the levels of SHBG are high, the level of free testosterone in plasma will be lower. In addition, Simon et al. (26) reported that oral oestrogen reduced both luteinizing hormone (LH) and follicle-stimulating hormone, thereby lowering total testosterone levels and reducing ovarian synthesis (26,31). Warnock et al. (14) also noted that birth control pills could lower the levels of testosterone as a result of the exogenous oestrogen found in birth control pills, which can further reduce LH and hinder ovulation (41,42). As such, the ovarian release of oestrogen is suppressed, and as a result, sexual libido is also affected. However, the levels of SHBG can be reduced using testosterone replacement therapy, which works by raising the levels of free testosterone and potentially decreasing potential signs and symptoms of HSDD (43,44). The next section elaborates on how hormonal influence affects FSD and contributes to HSDD in women.

## HORMONAL INFLUENCE AND ANDROGEN DEFICIENCY

**Figure 5:**
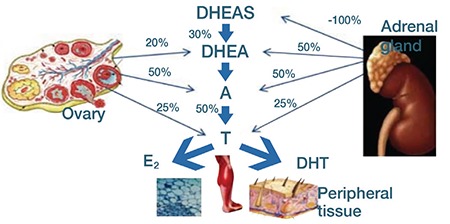
Production of androgens in the adrenal glands, peripheral tissues, and in the ovaries (45)

In women, androgens are C19 steroids generated from cholesterol, where the main sources of release are from the adrenal glands, peripheral tissues, and the ovaries. Figure 5 shows steroidogenesis of androgens in women. Androgens are released from peripheral tissues such as cutaneous, muscle, and adipose tissues. Figure 1 shows that testosterone (T) represents the final product in the androgen pathway and it results from the conversion of androstenedione (A) present in plasma. Half of the androgens come from the ovaries, 25% of the androgens are produced in the adrenal glands, and the other 25% comes from the conversion of androstenedione in peripheral tissues. In addition, the principal precursor of both androstenedione and testosterone androgens is dehydroepiandrosterone (DHEA), half of which is produced in the adrenal glands and 20% is generated from the ovaries; 30% is derived from dehydroepiandrosterone sulphate (DHEAS) that circulates in the blood. During post-menopause, DHEA, which is the main source of androgens, experiences an up to 60% decline resulting in hypoandrogenism, which can affect the normal sexual response in women (45).

As noted from the ageing factors associated with FSD, women can experience the effcets before and after menopause as a result of androgen hormone deficiency (46). Long before menopause, and specifically from the second half of the pre-menopausal years when a woman is aged between 30 and 50 years, the development of androgen hormones reduces from the ideal rate observed during puberty and up to the late 20s or early 30s (47,48). However, from the mid-30s, the normal activities of the ovaries reduce, and the process of ovulation becomes irregular.

**Figure 6:**
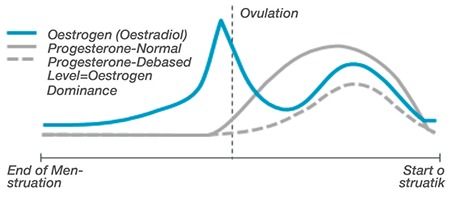
Oestradiol and progesterone cycle-dependent variations are showing oestrogen dominance (49)

As shown in Figure 6, in irregular ovulation cycles, there is less progesterone release, and in cycles where there is no ovulation, there is no release of progesterone (49). As such, as the levels of progesterone start to fall, the menstrual cycle becomes shorter and the lack of progesterone results in a hormonal imbalance where there is oestrogen dominance. The oestrogen dominance is shown in Figure 3, in relation to progesterone levels that are lower than normal among pre-menopausal women (49). Some of the symptoms linked to increased production of oestrogen at this age include depressive mood and anxiety. As an individual transitions into menopause (perimenopausal age), the irregular release of androgen hormones become longer, and women may have reduced sexual desire for prolonged months because they receive irregular menstrual cycles (50,51,52). At the age of 50 years, most women experience a significant reduction in the amounts of androgen, while the values for testosterone and oestrogen reach their minimum levels (53,54,55).

The process is characterised by the loss of androgen hormones with the situation reported to be worse in persons with hypopituitarism, bilateral oophorectomy, and Addison’s disease. Even so, the natural development of menopause can also result in reduced production of androgens (56,57). In most cases, androgen deficiency is difficult to identify, and most women correlate their reduced sexual desires with lifestyle issues or psychological distress as opposed to biologic changes in their bodies (58). Some of the experiences can result in an inexplicable lack of energy, tiredness, low self-motivation, disturbed sleep, a complete lack of sexual desire, and low self-esteem or poor general well-being (59,60). Low levels of androgens in women and reduced sexual desire can be diagnosed by examining levels of SHBG and testosterone because initial findings reported from women that have undergone surgery are as elaborated below.

## OOPHORECTOMY, HYSTERECTOMY, AND HSDD

Even if the changes in hormone profile among young women who have undergone hysterectomy and oophorectomy might not entirely affect sexual expression, the increased prevalence of HSDD in young women compared with pre- and post-menopausal women is a strong indicator for the affect of hormonal levels on sexual desire (61,62,63). The age-associated reduction in androgen hormones parallels the age-linked increase in HSDD among women, mainly in those who have reached natural levels of menopause with low sexual desire compared with pre-menopause women, further indicating the central role that hormones play in HSDD (64,65). As discussed earlier, low levels of oestrogen are largely associated with dyspareunia and vulvovaginal mucosa changes, a move that can contribute to reduced sexual desire among affected women (46,47).

In past studies, women who have undergone oophorectomy have shown to have associated low levels of sexual desire and increased distress or poor overall well-being. One study found lower levels of androgen hormones in healthy pre-menopausal women who reported having low sexual desire compared with women without a similar problem (66). The marked decline in low levels of testosterone after surgery has been linked to low sexual desire (67,68), because most studies have focused on safety, efficacy, and testosterone-route therapy to treat reduced sexual desire. Women who undergo bilateral oophorectomy experience a decline in testosterone between 40% and 50% from pre-surgical levels, and reduced libido between 30% and 50% (69,70). In addition to surgical procedures, a number of medical factors can also affect hormonal levels in women and contribute towards HSDD as discussed in the next section.

## MEDICAL FACTORS LINKED TO HSDD

A number of studies have also found a positive relationship between hypersexuality and medical factors. Some researchers reported that some treatments and medical conditions could negatively affect sexual desire among women. Table 1 summarises some diseases that have possible negative impacts on sexual libido. Medical interventions and diseases can change the physiology of sexual response both peripherally and centrally (71,72). Moreover, the presence of sexual disorders, including loss of sensitivity and pain, can trigger negative responses that can make such women lose interest in sexual expression (73).

Besides the chronic conditions that contribute to HSDD, Table 2 lists some common medicines reported to cause reduced sexual urge among women. For example, drugs that give healing benefits for diseases may negatively impact on sexual response among women (77). In most gynaecologic conditions, oral contraceptives are often used together in pregnancy prevention. For years, the combination and type of progestin and oestrogen have closely been reported in dealing with benign gynaecologic diseases and pregnancy prevention (78,79). However, notwithstanding the existing literature findings, the impact that these drugs have on women’s sexual changes still remain controversial (80).

Furthermore, there is increased connection between the oral contraceptive prescription in some women with vulvar vestibular pain. In patients with depression, serotonin-norepinephrine reuptake inhibitors (SNRI) and selective serotonin reuptake inhibitors (SSRI) medications are commonly prescribed antidepressants, although they commonly result in adverse events, including arousal difficulties, absent orgasm, delayed orgasm, and decreased desire. However, there continue to be few outcome studies evaluating the most effective agents in the management of FSD (80). The next section discusses some treatment approaches in the management of hormone-induced HSDD among women.

## TESTOSTERONE TREATMENT AND DESIRE

Poor awareness of FSD and the complex issues linked to HSDD development have largely reduced the formulation and research of therapeutic interventions for persons with low sexual desire (81). Several studies have been undertaken to assess the impact that sex hormones (androgens) have in HSDD management among affected women in menopause (82,83). Nevertheless, a proper understanding of the pathophysiology and physiology has triggered positive research progress in both pre- and post-menopausal female populations. The research process has also been encouraged by the need to have appropriate exclusion and inclusion criteria for FSD in clinical research using better analytical tools to examine primary outcome measures suitable in medication interventions (84,85,86).

In most cases, hormone therapy (using oestrogen alone) as indicated in oestrogen-progestin therapy (OPT) is widely used among menopausal women that have an intact uterus. Thus, the use of EPT is limited to women who report early symptoms (mainly hot flashes) as the first line of defence throughout the menopausal transition phase (80). Local and systemic use of oestrogen alone (OT) or with EPT has been reported as being an effective intervention in suppressing symptoms of vulvovaginal atrophy. The intervention has been reported to improve the sexual life of affected populations as a result of better lubrication (82,86). However, despite the reduction in dyspareunia, some women with FSD have been reported to be unresponsive because the OPT/OT does not have a consistent effect on the increase in sexual activity or desire, mainly among women grouped under surgical menopause (87,88).

Greenblatt et al. (89) conducted a randomised clinical trial and found that low sexual desire responded highly effectively to androgen therapies (AT). The authors also pointed out that low sexual desire responded even better to a combination of OT/AT, as opposed to using OT alone in ovariectomised women. Since this research, several studies have also demonstrated that androgens have an important role to play in terms of improving arousal and suppressing the negative impacts of FSD among women who have attained menopause. However, most studies have been based on supra-physiologic doses of hormone administration with testosterone (90).

Some Cochrane reviews have recently explored the risks and benefits of therapy, in addition to OPT+ OPT alone for both pre-menopausal and post-menopausal women where researchers included 35 studies with about 4800 participants. Most of the trials, which had several therapy regimens (including subcutaneous implants, intramuscular injections, gels or transdermal patches, and oral tablets), recruited only post-menopausal women–both surgically and naturally menopausal–with low sexual desires. The medium intervention period was six months and ranged from one and half months to 24 months. A pooled approximation from the examined clinical trials indicated that by adding therapy to hormone therapy, the women’s sexual response improved and led to improved satisfaction in sexual incidents among post-menopausal women. These beneficial effects were reported and measured for coital frequency, desire, responsiveness, and sexual activity (91,92).

However, some adverse effects were also reported, including increased cases of acne and excess hair growth and reduced levels of high-density lipoprotein. When this intervention was discontinued, the outcome was similar for both groups. Among the perimenopausal women, however, there was insufficient evidence about the efficacy of this treatment or for additional outcomes that were explored, including body composition, cognition, menopausal symptoms, fatigue, and well-being. Another study examined the effect of transdermal T patch in post-menopausal women with HSDD. The randomised, double-blind, and placebo-controlled research was evaluated over a 24-week period with over 1200 participants who were surgically menopausal with HSDD and received affiliated oestrogen therapy (93,94,95).

The baseline research reported that women had three episodes of increased sexual desire during the first four weeks when the 300 μg T patch was used compared with a single satisfying event among the placebo group. Nonetheless, a 450 μg T patch had no benefits compared with a non-intervention placebo group, indicating the absence of dose-response from additional T patch intervention (94). Besides increased sexual activity, there were also improvements in domains of sexual functions among women who received T patches and those from the placebo group including pleasure, orgasm, distress, sexual self-image, responsiveness, concerns, and desire. As a result, there was an increase in sexual episodes with the use of the therapy compared with placebo (95). As such, the use of hormone therapy shows significant improvement of sexual response and suppression of HSDD among women with the condition.

In conclusion FSD, grouped either as HSDD, has been shown to be a highly prevalent sexual condition, which has negative outcomes on the women’s well-being and sexual life. Despite this, HSDD remains a common underdiagnosed condition by physicians, and it also has few treatment regimens. Even so, a number of factors have recently converged to create a suitable shift toward greater awareness and attention. For instance, increased focus on hypoactive sexuality as a topic in menopause research has increased interest in the field of female fertility and further changed the previous focus on the topic. The shift has also resulted from a change in societal perception about women’s privilege to a healthy sexual lifestyle, even if most post-menopausal women still possess the perception that sexuality is a taboo subject.

Today, the increased search for effective pharmacologic agents to manage various biologic causes of HSDD is a primary indicator of the strong forces that are currently initiating more attention on the topic among physicians and researchers. Most studies have now weighed in by including FSD as a disease area that deserves unique and separate research focus. In addition, a number of pharmacologic agents have been designed to target HSDD and are in various stages of clinical trials. However, the field still continues to face some hurdles including a lack of information, confusion over medications and management, and the discomfort associated with addressing the subject of sexuality. Therefore, the value of the current review will be enhanced by addressing the current barriers to the topic and committing more resources to understanding the role that hormones play in HSDD.

## Figures and Tables

**Table 1:**
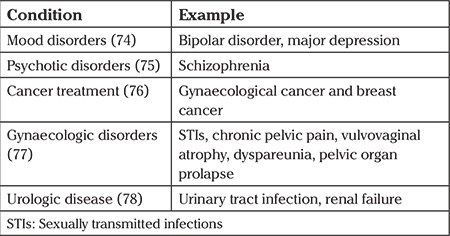
Long-term medical conditions that lead to hyposexuality in women

**Table 2:**
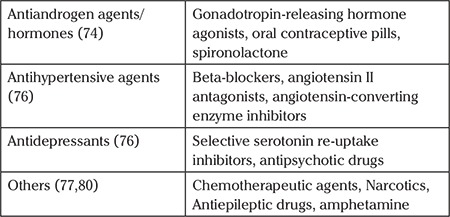
Some medications that affect sexual desire among women
